# Supplementary material for: Genome-Wide Identification and Analysis of Collar Region-Preferential Genes in Rice
Source: Plants (Basel). 2023 Aug 16;12(16):2959. doi: 10.3390/plants12162959 (PMC10458737; doi:10.3390/plants12162959)
Supplement: Supplementary file 1 [file plants-12-02959-s001.zip › Supplementary figure caption.pdf]

Figure S1. Venn diagram of functionally characterized CRPGs.

Figure S2. KEGG enrichment analysis for 657 CRPGs.

Figure S3. MapMan analysis for 657 CRPGs. Results of mapping 657 CRPGs to (a) metabolism overview and (b) regulation overview.
