# Supplementary figures and images for: Genome-Wide Identification and Analysis of Collar Region-Preferential Genes in Rice
Source: Plants (Basel). 2023 Aug 16;12(16):2959. doi: 10.3390/plants12162959 (PMC10458737; doi:10.3390/plants12162959)

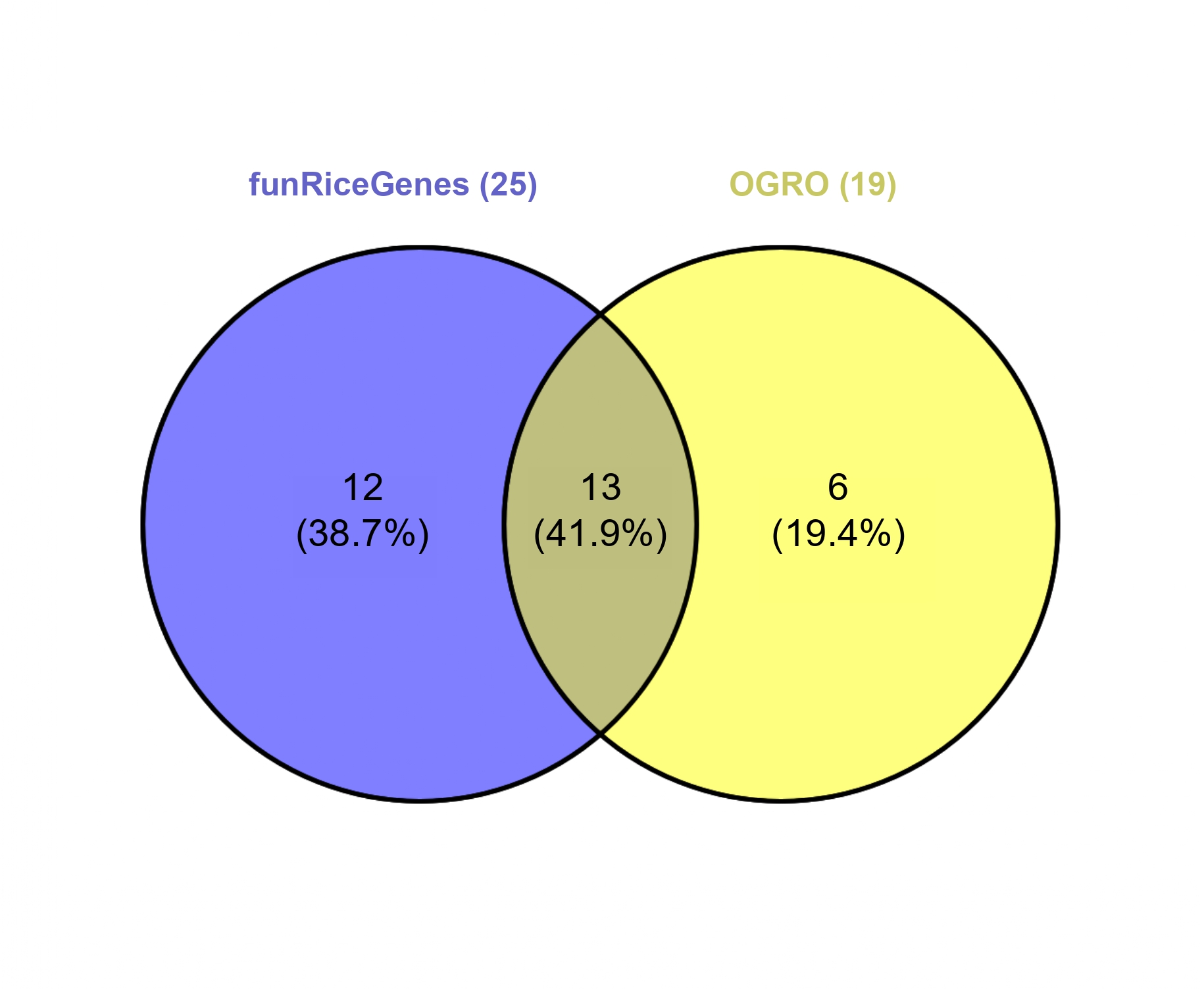

Supplement: Supplementary file 1 [file plants-12-02959-s001.zip › Figure S1.jpg]

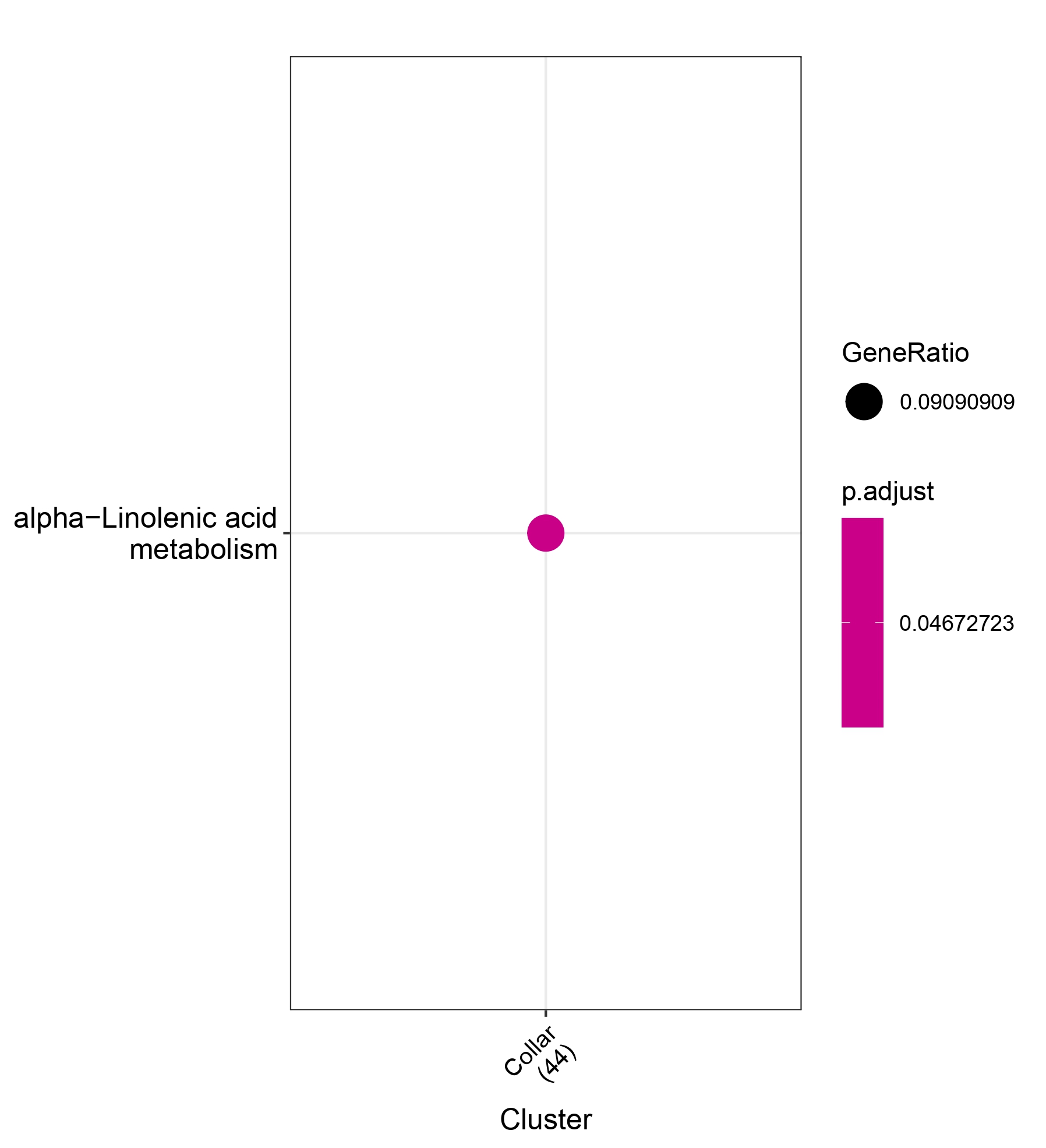

Supplement: Supplementary file 1 [file plants-12-02959-s001.zip › Figure S2.jpg]
